# M1 macrophages – unexpected contribution to tumor progression

**DOI:** 10.3389/fimmu.2025.1638102

**Published:** 2025-07-31

**Authors:** Olga V. Kovaleva, Madina A. Rashidova, Vasiliy V. Sinyov, Olga S. Malashenko, Alexei Gratchev

**Affiliations:** ^1^ Institute of Carcinogenesis, N. N. Blokhin National Medical Research Center of Oncology, Moscow, Russia; ^2^ Center for Molecular and Cellular Biology, Skolkovo Institute of Science and Technology, Moscow, Russia

**Keywords:** macrophage, inflammation, tumor, innate immunity, immunotherapy, ADCC, ADCP

## Abstract

The anti-tumor role of the immune system has long been associated with interferon-γ-mediated activation of immune cells and their ability to recognize and eliminate transformed cells. Fundamental principles of tumor immunoediting describe a dynamic interplay between the immune system and neoplastic cells, wherein immune pressure can paradoxically shape tumor evolution. Within this context, macrophages, natural killer cells, and T lymphocytes are central effectors of anti-tumor immunity. Traditionally, macrophages exhibiting M1 phenotype are characterized by high cytotoxic potential and considered important contributors to tumor eradication. In contrast, M2-polarized tumor-associated macrophages are associated with immune suppression and tumor progression. However, recent evidence challenges this binary paradigm. It is increasingly evident that M1 macrophages, while initially exerting anti-tumor effects, can also promote tumor progression by applying sustained cytotoxic pressure that selects for more malignant and immune-resistant tumor clones. This phenomenon represents an unexpected and overlooked contribution of cytotoxic macrophages to tumor progression. In this review, we examine the complex, context-dependent function of M1 macrophages and reassess current strategies aimed at enhancing their cytotoxicity. While such approaches may offer short-term benefits, they risk driving clonal selection of aggressive, immune-evasive tumor cells. Therefore, we propose a paradigm shift: instead of promoting M1 polarization alone, therapeutic strategies should consider the broader consequences of macrophage–tumor interactions. A nuanced understanding of macrophage plasticity and tumor dynamics is essential for designing effective immunotherapies. Recognizing the paradoxical role of M1 macrophages is critical to avoiding unintended support of tumor evolution and improving treatment outcomes.

## Introduction

Experimental evidence confirms that the tumor stroma is an essential component of malignant neoplasms and plays a critical role in disease progression. It is primarily composed of various mesenchymal cell types, including fibroblasts, endothelial cells, and a broad spectrum of immune cells (1). In the early stages of tumor development, immune cells within the stroma may exert anti-tumor effects. However, as the tumor evolves, these cells often undergo phenotypic shifts toward immunosuppressive profiles, ultimately promoting tumor growth and dissemination. The immune infiltrate within tumors is highly heterogeneous, comprising T lymphocytes, neutrophils, macrophages, myeloid-derived cells, natural killer (NK) cells, and dendritic cells. This dynamic and complex cellular network highlights the intricacies of tumor immunology and underscores the importance of immune regulation in cancer progression (2).

At the onset of carcinogenesis, immune response is activated, specifically aimed at suppressing tumor growth and eradicating malignantly transformed cells. Macrophages, are the crucial players in this defense mechanism. During this early stage of tumor development, the tumor cells express a broad spectrum of protein and non-protein antigens. These antigens, can be recognized by macrophages, and include well-documented tumor-associated proteins from the MAGE, GAGE, and BAGE families, glycoproteins such as gp100, NY-ESO-1, HER-2/neu, MUC1, WT-1, and some others (3). Despite this antigenic diversity, the intrinsic heterogeneity of the tumor often results in a variable immunogenic profile among its cells. Not all malignant cells demonstrate a high level of immunogenicity, complicating the ability of immune system to uniformly detect and eliminate them. The tumor survival strategies are sophisticated, involving the emergence and selective proliferation of cells with diminished or absent expression of these tumor antigens. These cells effectively evade immune surveillance by camouflaging themselves within the normal cellular landscape of the body. This evasion is not just a passive process but a dynamic adaptation that challenges the capacity of the immune system to maintain systematic surveillance and effective tumor control.

In addition to passive mechanisms of evasion from immunological surveillance, tumor cells are capable of activating more direct methods. During tumor development, due to mutagenesis, tumor cells start to express both surface and soluble molecules that modify the activation characteristics of immune system cells. For instance, the expression of cytotoxic T-lymphocyte-associated antigen 4 (CTLA-4) on the surface of a tumor cell results in the inhibition of the cytotoxic activity of T-cells (4). Malignant cells produce interleukins like IL-6, IL-13, IL-2, and IL-12, which shift cytotoxic macrophages to an immunoregulatory phenotype (5). These macrophages, in turn, begin to produce factors that promote tumor progression, such as transforming growth factor beta (TGF-β) and vascular endothelial growth factor (VEGF) (6). Therefore, within the tumor microenvironment, cells exhibiting an immunosuppressive phenotype develop, thereby promoting the progression of the disease. These cells not only subvert immune detection but also reprogram the local immune environment to support tumor cells growth and proliferation, thereby advancing the complexity and severity of the tumor.

The cells of the tumor microenvironment are categorized into two groups based on their functions. The first group includes cytotoxic cells (dendritic cells, pro-inflammatory macrophages (M1), CD8+ and CD4+ T-lymphocytes, B-lymphocytes, and NK cells), which contribute to the suppression of tumor progression. In contrast, regulatory T-cells (Treg) and immunosuppressive macrophages (M2) reduce the effectiveness of the immune response by limiting the activation of lymphocytes and specific immune reactions. These dynamics illustrate the complex interplay within the tumor microenvironment, where various cell types either combat or facilitate the progression of the tumor, significantly influencing the overall outcome of the disease.

Macrophages are multifunctional cells whose phenotype develops under the influence of the surrounding cytokine environment. In the context of a tumor, due to the action of cytokines and growth factors produced by tumor cells, an immunosuppressive phenotype of macrophages - M2 is developed. These tumor-associated macrophages (TAMs) contribute to the progression of the tumor and increase its malignant potential (7). Furthermore, it is known that increased infiltration of M2 in the tumor stroma is a marker of poor prognosis for most solid tumors (8). This relationship highlights the critical role of the tumor microenvironment in shaping the behavior of TAMs, directly impacting the aggressiveness and clinical outcomes of the disease.

## Macrophage features and functions

These discoveries have prompted a deeper exploration into macrophage biology, particularly their functional diversity in different pathological settings. In cancer, the dual nature of macrophage phenotypes has become a focal point of research, as their influence over tumor progression or suppression hinges on the microenvironment. While M1 macrophages demonstrate cytotoxic activity capable of targeting tumor cells, their presence in certain contexts can paradoxically contribute to tumor evolution by exerting selective pressure. This underscores the crucial need for therapeutic strategies that carefully consider the full spectrum of macrophage functions.

The last decade has seen a significant shift in our understanding of the origins of tissue macrophages. Studies using animal models have revealed that most tissue macrophages actually form during embryonic development. These resident macrophages typically originate from hematopoietic precursor cells located in specialized sites such as the yolk sac, fetal liver, and bone marrow. It was observed that while these embryonically derived macrophages are maintained throughout life in some tissues, in others, particularly under conditions of inflammation or as the organism ages, macrophages differentiated from circulating monocytes become the predominant population (9, 10).

The implications of these findings are profound, indicating that macrophages are not a uniform cell population but are instead highly diverse. The local microenvironment significantly influences their phenotype and functions, leading to a complex landscape of macrophage activity within different tissues. This variability is crucial for understanding the role of macrophages in health and disease, including their involvement in tissue repair, inflammation, and immune surveillance. This evolving paradigm enhances our ability to target specific macrophage populations for therapeutic interventions in diseases such as cancer, autoimmune disorders, and chronic inflammatory conditions.

### Macrophage dichotomy

There are at least two principal types of macrophage activation within the immune system: classical (M1) and alternative (M2) (11, 12). The classical or pro-inflammatory phenotype is initiated primarily in response to cytokines secreted by Th1 type T-cells, such as interferon-gamma (IFN-γ) and tumor necrosis factor (TNF). Additionally, components of bacterial cell walls like lipopolysaccharide (LPS) and muramyl dipeptide (MDP), as well as other pathogen-associated molecular patterns (PAMPs), also trigger M1 activation (13). M1 macrophages are integral to the inflammatory process, not only participating actively in immune defense mechanisms but also possessing cytotoxic capabilities that can directly target and destroy tumor cells (14). They are key producers of a wide array of effector molecules and pro-inflammatory cytokines.

### M1 macrophages: inflammatory and cytotoxic function

The classical activation pathway endows M1 macrophages with enhanced expression of class II major histocompatibility complex receptors (HLA-DR) and inducible nitric oxide synthase (iNOS), both critical for their role in antigen presentation and microbial killing (15, 16).

Additionally, markers commonly associated with M1 macrophages include CD11c, CD86, and the phosphorylated form of STAT1 (pSTAT1). pSTAT1 acts as a transcription factor that regulates genes essential for the cytotoxic functions of macrophages, influencing their ability to respond to infectious threats and malignantly transformed cells effectively. Through these mechanisms, M1 macrophages contribute significantly to the body’s first line of defense, orchestrating both innate and adaptive immune responses (16).

While M1 macrophages were initially known as activated macrophages (17), M2 macrophages described decades later (18) have gained more attention due to their role in supporting tumors. In response to cytokines secreted by Th2 type T-cells (IL-4, IL-13, IL-33, IL-10, IL-21), as well as other mediators such as TGF-β, vitamin D3, and glucocorticoids, the immunosuppressive M2 phenotype of macrophages is established (11). These M2 macrophages are crucial for maintaining tissue homeostasis and possess notable anti-inflammatory functions, which are essential in tissue repair and regeneration (13). Additionally, they have a significant role in promoting tumor growth by creating an environment that supports tumor survival and expansion. The development of the M2 macrophage phenotype is largely mediated through the activation of the transcription factor STAT6 (19), which orchestrates a network of genes responsible for their immunosuppressive and tissue repairing functions.

### M2 macrophages: immunoregulatory and pro-tumor functions

M2 macrophages are characterized by an enhanced production of anti-inflammatory cytokines such as IL-10 and TGF-β, and growth factors like VEGF, which are crucial for angiogenesis and tissue repair. These macrophages also exhibit a reduced secretion of IL-12, supporting their role in damping inflammatory responses. The expression of surface markers like mannose receptor-1 (CD206) and scavenger receptors (CD204 and CD163) is markedly increased in M2 macrophages, aiding in the clearance of debris and dead cells, thereby maintaining homeostasis (20, 21).

M2 macrophages are not a uniform population but consist of distinct subtypes—M2a, M2b, M2c, and M2d—each induced by different stimuli and performing specific roles in immune regulation and tumor progression (22). M2a macrophages are generated in response to IL-4 and IL-13 and are primarily involved in tissue repair and fibrosis and contribute to tumor dissemination (23). M2b macrophages are induced by immune complexes in combination with TLR agonists or IL-1β. These cells display a mixed cytokine profile, simultaneously producing pro-inflammatory (e.g., IL-1β, TNF) and anti-inflammatory (e.g., IL-10) mediators. Their immunoregulatory nature allows them to suppress adaptive immune responses while maintaining chronic inflammation that favors tumor development (23). M2c macrophages arise under the influence of IL-10, TGF-β, or glucocorticoids and are strongly immunosuppressive. They are involved in matrix deposition, clearance of apoptotic cells, and promotion of tumor tolerance. Their high expression of CD163 and MerTK receptors aligns them closely with the phenotype of tumor-associated macrophages found in various cancer types (23, 24). M2d macrophages, often equated with tumor-associated macrophages (TAMs), are induced by IL-6 and adenosine signaling within the tumor microenvironment. They are potent promoters of angiogenesis, mainly through VEGF production, and they suppress anti-tumor immune responses by inhibiting cytotoxic T-cell function and promoting regulatory T-cell expansion (22).

## Cytotoxic functions of macrophages

### Contact-independent mechanism

The cytotoxic activity of macrophages enables these cells to destroy tumor cells through both direct and indirect mechanisms. The primary mechanisms of direct cytotoxic activity include phagocytosis, the production of pro-inflammatory cytokines, and mediators of inflammation such as nitric oxide and reactive oxygen species, which trigger processes of programmed cell death in the target cells (25). Additionally, macrophages attract cells of the adaptive immune system, such as T-cells, to the site of inflammation.

The mechanism of macrophage cytotoxic activity can be classified into contact-dependent and contact-independent interactions with the target cell. The initiation of contact-independent cytotoxic activity by macrophages primarily occurs in response to soluble factors (cytokines) produced by T-lymphocytes following interactions of T-cells with antigen-presenting cells or mitogens, such as phytohemagglutinin (PHA) and concanavalin A (Con A) (26). Well-known cytokines include Macrophage Activation Factor (MAF) and Macrophage Migration Inhibitory Factor (MIF) produced by T-cells regardless of contact with the tumor cell (27). The interaction with cytokines leads to macrophage activation. Cytotoxic activity is conducted without direct physical contact through secreted soluble factors by macrophages, such as cytokines, chemokines, as well as reactive oxygen and nitrogen species, which lead to the death of the target cells (28).

This complex interplay not only facilitates the elimination of tumor cells but also significantly impacts the microenvironment by modulating inflammatory responses and orchestrating the recruitment and activation of other immune cells. This nuanced role of macrophages highlights their importance in both innate and adaptive immune responses, making them a crucial target for therapeutic strategies aimed at enhancing anti-tumor immunity.

### Contact-dependent mechanism

The contact-dependent mechanism can occur via antibodies bound to the surface of the target cell (antibody-dependent cellular cytotoxicity, ADCC), as well as without the involvement of antibodies. In the context of anti-tumor immune response, macrophage-mediated ADCC plays a central role in cytotoxic activity. Moreover, this mechanism mediates the action of many immunotherapeutic drugs based on monoclonal antibodies (mAbs) (29, 30). ADCC by macrophages is primarily carried out through phagocytosis. This process is initiated by the binding of Fc receptors on the surface of macrophages to antibodies on the surface of malignantly transformed cells. This mechanism can be enhanced by the action of certain cytokines and mAbs. For instance, it is known that IL-15, IL-21, IL-18, IL-2, and antibodies to CD137, CD96, TIGIT, KIR, PD-1 possess this activity (31). There is evidence that cytokines and mAbs act synergistically in the context of anti-tumor therapy. For example, IL-15 enhances the efficacy of mAbs against CD20 and CD52 (32). It is known that the number of engaged Fc receptors on the surface of macrophages directly correlates with the effectiveness of ADCC in the context of tumor cells (33).

The data presented in the scientific literature about the mechanism of antibody-independent cytotoxic activity of macrophages are fragmented. It is known that this process also requires opsonization of the target cell. In this case, complement factors act as opsonins. The opsonization of tumor cells with the complement component C3, along with the generation of pro-inflammatory mediators C3a and C5a, activates the cytotoxic activity of macrophages. The C3 components of the complement on the surface of the tumor cell are recognized by macrophages through complement receptors CR3 and CR4 (CRs), which results in increased FcγR-mediated phagocytic activity (34). There is evidence that the C9 factor plays an important role in complement-mediated cytotoxic activity of macrophages in the context of non-small cell lung cancer (35).

Antibody-independent cytotoxic activity of macrophages can be enhanced by the action of IFN-γ, bacterial products such as LPS, MDP, and other PAMPs. The action of IFNγ is mediated by the phosphorylation of the transcription factor STAT1, which initiates the transcription of about 200 genes, most of which are associated with inflammation (36). In response to the interaction of TLRs on the surface of macrophages with PAMP, a cascade of reactions is triggered, leading to an increase in the cytotoxic activity of the immune cell. For instance, LPS, by binding to TLR4, initiates a cascade of reactions that activate the transcription factor NFkB, resulting in the activation of transcription of genes for pro-inflammatory cytokines such as TNF, IL-1β, IL-6, IL-12, IL-27, as well as nitric oxide synthase (NOS2), and others (37). The bacterial cell wall component MDP activates macrophages by binding to another receptor, NOD2. This interaction also activates NF-κB, subsequently enhancing the cytotoxic potential of the effector cell.

These insights into the antibody-independent cytotoxic mechanisms underscore the sophisticated nature of macrophage activation and their crucial role in innate immunity. By harnessing such pathways, macrophages are capable of directly combating pathogenic and cancerous cells without the direct need for antibody mediation, marking them as key players in the body’s defense system against a variety of threats.

### Macrophage-derived anti-tumor factors

Thus, the primary function of macrophages in the context of malignant neoplasms is anti-tumor. Recruited monocytes primarily differentiate into M1 macrophages and produce a range of inflammatory mediators that activate the immune response. Some of these mediators initiate feedback loops. For example, IL-12 produced by M1 macrophages stimulates NK cells and dendritic cells to secrete IFN-γ, which enhances the cytotoxic potential of macrophages, including an increase in the production of reactive oxygen species and nitric oxide (NO). These compounds lead to the activation of apoptosis in the target cell. One of the primary targets of reactive oxygen species within cells, including malignantly transformed ones, are lysosomes. Oxidation causes destabilization of the lysosomal membrane, leading to the release of lysosomal enzymes and damage to the cell. In response, the cell activates the process of autophagy as a defense mechanism; however, prolonged oxidative stress leads to what is known as autophagic cell death, which is currently classified as a type of programmed cell death (38).

Programmed cell death processes in tumor cells are also triggered in response to other inflammatory cytokines such as TNF, IL-1β, MCP-1 (monocyte chemotactic protein 1), and others. ADCP (antibody-dependent cellular phagocytosis) is accompanied by the presentation of tumor antigens to T-cells and the activation of an adaptive anti-tumor immune response. Activated lymphocytes proliferate, forming tumor-specific clones and infiltrating the tumor, thus forming an adaptive anti-tumor immunity (38). M1 macrophages can inhibit tumor development significantly through phagocytosis and the presentation of antigens on their surface, recruiting CD8+ T-cells and cytotoxic NK cells (39).

Recent studies have also highlighted the significant impact of intracellular molecules, such as microRNAs (miRNAs), on the polarization of macrophages. For instance, miR-720 is known to push M2 macrophages towards an M1 phenotype while simultaneously inhibiting their phagocytic activity, suggesting a complex regulatory mechanism that balances pro- and anti-inflammatory responses (40). Similarly, miR-127 enhances the expression of pro-inflammatory cytokines like IL-6 and IL-1β, suggesting its potential role in promoting an M1 phenotype, which is geared towards fighting infections and tumor cells (41). Moreover, miRNAs such as miR-23a/27a/24–2 are actively involved in reprogramming macrophages towards an M1 phenotype, thereby supporting anti-tumor activity. miR-23a, by interacting with the NF-κB pathway inhibitor A20, not only promotes the expression of inflammatory cytokines but also disrupts the immunosuppressive signaling pathways, typically prevalent in M2 macrophages, through inhibition of the JAK1/STAT6 pathway. miR-27a exerts similar effects by targeting regulatory factors like IRF4 and the PPARγ receptor, further demonstrating the intricate network of gene regulation involved in macrophage polarization (42).

These mechanisms illustrate the critical roles that M1 macrophages play not only in direct tumor cell elimination but also in orchestrating a broader immune response against tumors. Their ability to present antigens and recruit other immune cells underscores the importance of macrophages in the development of effective anti-cancer strategies, highlighting potential therapeutic targets for enhancing anti-tumor immunity.

### Macrophage-based therapeutic approaches

Based on these observations, numerous therapeutic approaches have been developed to reprogram pro-tumoral M2 macrophages into inflammatory M1 cells within the tumor microenvironment. One approach involves the use of pattern-recognition receptor agonists: TLR7/8 ligands (43), TLR3/5/9 agonists delivered by ROS-inducing micelles (44), ferritin (45) or liposomal systems that trigger NF-κB/IRF cascades in F4/80^+^ cells inside the tumor. These treatments promote the expression of iNOS and IL-12, leading to enhanced antitumor immunity in murine models (45–48). Another strategy relies on metabolic reprogramming of TAMs, including pH-responsive micelles or exosomes that silence STAT6 (49) and miR-155 conjugated graphene quantum dots (50) re-educating resident TAMs. Additional approaches include checkpoint-targeted and vesicle-based systems such as dual-inhibitor supramolecules (CSF-1R + SHP2), SIRPα-blocking magnetic nanoparticles (51), and hybrid nanovesicles that fuse M1-derived membranes with CD47-targeting modules (52).

Clinical translation of these approaches was, however, limited. In patients with melanoma, non-small cell lung cancer, and renal cell carcinoma, CSF-1R blockade—alone or in combination with CD40 agonists and nivolumab—failed to induce durable M2-to-M1 repolarization and resulted in low objective response rates (53, 54). Broad myeloid-targeted combinations, such as CSF-1R with CCR2/5 and CXCR2 inhibitors, were similarly ineffective, as compensatory immunosuppressive myeloid populations rapidly re-emerged (55). Moreover, the multi-kinase CSF-1R inhibitor pexidartinib caused off-target depletion of dendritic cells and liver toxicity when combined with durvalumab, yielding only limited partial response rate in advanced colorectal and pancreatic cancers (56).

## Pro-tumor function of M1 macrophages

### Inflammatory factors

The cytotoxic activity of type I macrophages may paradoxically facilitate tumor progression. Reactive oxygen species (ROS), nitric oxide (NO), and a spectrum of pro-inflammatory cytokines such as IL-6, TNF, and IFN-γ can exert mutagenic effects on tumor cells and their surrounding microenvironment (57, 58). These inflammatory mediators, while intended to combat tumor cells, can unintentionally promote genetic mutations that lead to enhanced tumor survival and adaptation. Furthermore, certain chemokines produced by cytotoxic macrophages serve as chemoattractants for regulatory T-cells (Tregs), which are known to suppress anti-tumor immune responses and thus facilitate tumor progression (59).

TNF, in particular, plays a critical role in promoting tumor angiogenesis, proliferation, invasion, and metastasis (60). This cytokine activates the NF-κB signaling pathway within tumor cells, leading to increased tumor cell survival and proliferation. Notably, TNF exposure results in a loss of gp100 protein expression in melanoma cells, while simultaneously elevating levels of the neurotrophin receptor (NGFR) (61). Since gp100 is a recognized target for immune attack and NGFR is linked with tumor aggressiveness, this shift could lead to decreased immune surveillance and increased tumor malignancy. Moreover, NGFR’s role in inactivating the tumor suppressor gene p53 further underscores its contribution to tumor growth and resistance to cell death (62).

TNF also attracts endothelial cells, fibroblasts, and pericytes to the tumor site, facilitating the formation of a supportive tumor microenvironment that is conducive to further growth and spread. The production of matrix metalloproteinases by cytotoxic macrophages, often seen in high levels within the tumor microenvironment, aids in breaking down extracellular matrix barriers, thus enabling tumor invasion and metastasis (63). Additionally, the presence of IFN-γ induces macrophages to express indoleamine 2,3-dioxygenase (IDO), which suppresses cytotoxic T-lymphocyte activity, further dampening the immune response against tumor cells (64, 65).

The enduring M1/M2 paradigm maintains that while M1 macrophages are typically anti-tumoral, M2 macrophages generally promote tumor growth. This dichotomy underscores the dualistic nature of macrophage function in cancer biology (66). Current research continues to explore macrophage reprogramming strategies, aiming to convert pro-tumoral M2 macrophages back into anti-tumoral M1 types, thus enhancing the overall effectiveness of anti-cancer therapies (67). Nevertheless, emerging studies challenge this binary classification, revealing scenarios where M1 macrophages unintentionally support tumor growth, highlighting the complexity and dynamic behavior of these immune cells within different tumor contexts. This evolving understanding necessitates a more nuanced approach in leveraging macrophages in cancer therapy, ensuring that interventions precisely target the multifaceted roles these cells play in tumor progression.

### Experimental evidence

For instance, it has been demonstrated that conditioned medium from M1 macrophages can stimulate the invasive capacity of pancreatic adenocarcinoma cells, as shown by increased migration and invasion of MiaPaCa-2 and HPAF-II cells in response to GM-CSF–polarized M1 macrophages derived from human blood monocytes (68). In hepatocellular carcinoma (HCC), exposure of monocytes to HCC-conditioned medium induced an M1-like phenotype that paradoxically promoted tumor growth *in vivo* by suppressing tumor-specific T cells; notably, this effect was reversed by PD-L1 blockade (69). Moreover, M1 macrophages generated by stimulation of U937 cells with IFN-γ and LPS were shown to enhance proliferation and invasion while reducing apoptosis in HepG2 and SMMC-7721 hepatocellular carcinoma cells (70). In a melanoma model it was demonstrated that the conditioned medium from M1 macrophages can stimulate the invasive capability of tumor cells through activation of the TNFR–NF-κB signaling pathway (71). It has also been shown that the conditioned medium from M1 macrophages promotes the proliferation of hepatocellular carcinoma cells via the NF-κB signaling pathway (72). Furthermore, the conditioned medium from M1 macrophages has been found to stimulate the proliferative ability of gastric cancer cells (73). Recently, it has been shown that M1 macrophages enhance the survival and invasion of squamous cell carcinoma cells of the oral mucosa by activating ErbB2 (74). **Table 1** provides an overview of studies discussed.

**Table 1:** Summary of studies demonstrating pro-tumorigenic effects of M1 macrophages.

Publication	M1-activation strategy	Cancer model tested	Pro-tumor read-out demonstrated
Salmiheima 2016 (68)	Human blood monocytes stimulated with GM-CSF	Pancreatic‐adenocarcinoma cell lines MiaPaCa-2, HPAF-II	Increased tumor-cell migration/invasion
Kuang 2009 (69)	Human monocytes exposed to HCC-conditioned medium.	Human HCC samples; HepG2 xenografts in NOD/SCID mice.	M1 suppressed tumor-specific T-cells and accelerated tumor growth *in vivo*; PD-L1 blockade reversed.
Xie 2016 (70)	U937 stimulated with IFN-γ, LPS	HepG2, SMMC-7721 HCC cells.	Increased proliferation, invasion; reduced apoptosis.
Kainulainen 2022 (71)	THP-1 stimulated with PMA, IFN-γ, LPS	Melanoma lines MV3, A375	Increased invasion.
Sharen 2022 (72)	THP-1 stimulated with PMA, IFN-γ, LPS.	Hepatocellular carcinoma cells HepG2, SNU-182	Increased proliferation, clonogenicity, radio/chemo-resistance.
Zhou 2018 (73)	THP-1 stimulated with PMA, LPS	Gastric-cancer lines BGC823, MKN28	Accelerated proliferation.
Lv 2022 (74)	THP-1 stimulated with PMA, IFN-γ, LPS.	OSCC lines SCC25, CAL27; nude-mouse xenografts.	Increased proliferation, colony-formation, migration/invasion
Podlesnaya 2022 (75)	THP-1 stimulated with PMA, IFN-γ, LPS.	Cell lines PC3 (prostate), H1299 (lung)	Resistance to macrophage killing, increased proliferation, migration
Kovaleva 2022 (76)	THP-1 stimulated with PMA, IFN-γ, LPS.	H1975 (lung), nude-mouse xenografts.	Increased proliferation *in vitro* and *in vivo*, increased tumor size with vascularization

The findings from these studies highlight a complex paradox where M1 macrophages, traditionally considered as anti-tumoral, can under certain conditions promote tumor progression. This phenomenon may be explained by the multifaceted nature of the cytokine and chemokine profiles secreted by M1 macrophages, which, while aimed at fighting infections and tumors, can inadvertently provide growth factors and survival signals to cancer cells. The local tumor environment also plays a critical role in dictating the effects of these signals, with certain cancer types possibly more predisposed to exploit the inflammatory milieu to their advantage.

These insights underscore the need for a deeper understanding of the tumor microenvironment and the interplay between immune cells and cancer cells. This knowledge is crucial for designing targeted therapies that can modulate the tumor-promoting effects of M1 macrophages or potentially harness their anti-tumoral capabilities more effectively. As research progresses, strategies may need to be tailored to not only enhance the cytotoxic functions of M1 macrophages but also mitigate their potential to support tumor growth, ensuring that therapeutic interventions are both precise and effective in combating cancer.

Previous research on the resident microbiome and tumor stroma prompted us to explore the potential pro-tumoral role of type 1 activated macrophages (M1). We hypothesized that under certain conditions M1 macrophages may contribute to tumor progression by cytotoxic pressure that selects more malignant, resistant tumor clones. To test this hypothesis, we created a unique *in vitro* model in which tumor cell lines of various origins (lung, prostate, kidney, breast) were repeatedly exposed to macrophages stimulated with the TLR4 ligand lipopolysaccharide (LPS), leading to the development of tumor cell sublines resistant to macrophage cytotoxicity (75). This model enabled us to characterize tumor cells that acquired resistance to macrophage cytotoxicity. Our findings revealed that these resistant sublines exhibited several features associated with increased malignancy, including accelerated proliferation, enhanced tumor growth *in vivo*, increased vascularization, and perineural invasion (76). Transcriptomic analysis further identified signaling pathways and gene expression changes potentially underlying this acquired resistance. While these results suggest that inflammatory macrophages may act as a selective pressure favoring immune-evasive and more aggressive tumor phenotypes, we acknowledge that this does not establish a direct mechanistic link between M1 macrophages and immune escape. These findings support the idea that pro-inflammatory macrophages may contribute to tumor evolution by selecting clones capable of resisting their cytotoxic effects, although further studies are needed to clarify the precise mechanisms involved in this process.

### Possible mechanisms

An analysis of the transcriptome of the derivative cell lines compared to the originals revealed the activation of various signaling pathways potentially involved in tumor progression, particularly the integrin-dependent signaling pathway and the TGF-β signaling pathway. Signaling pathways activated by the cytokine TGF-β regulate a large number of biological processes, such as cell division, migration, and differentiation, and their effects vary depending on the type of target cells and their microenvironment. One of the primary mechanisms of TGF-β action involves interaction with Smad proteins, leading to the regulation of numerous genes. On the other hand, TGFβ can activate the function of MAP kinases, specifically p38, through Smad-independent mechanisms. It is also noteworthy that transcriptomic sequencing revealed an increase in the expression of osteoprotegerin (OPG), also known as a member of the 11b superfamily of TNF receptors (TNFRSF11B). This receptor is a soluble protein whose main function is to inhibit TRAIL-induced apoptosis. For various solid tumors, an association of OPG content with tumor aggressiveness has been demonstrated (77–80). It has been shown that OPG secretion is mediated by the activation of two signaling cascades, namely p-38 and ERK1/2, which, in turn, are activated in response to the cytokine IL-1β produced by macrophages (81), which is consistent with our results.

These findings suggest that while M1 macrophages are typically considered anti-tumor, their activity can, under certain conditions, promote tumor progression by exerting selective pressure that favors the outgrowth of resistant and more aggressive clones. This paradox highlights the complex and context-dependent nature of macrophage–tumor interactions and underscores the need for nuanced approaches in cancer therapy that go beyond simple macrophage activation. Rather than broadly stimulate M1 functions, future strategies should aim to preserve their cytotoxic potential while minimizing the pro-tumoral effects of sustained inflammatory signaling. The specific signaling pathways responsible for this shift remain to be identified, but therapeutic targeting of downstream cytokine effects and modulation of the tumor microenvironment may help prevent the unintended promotion of tumor growth.

## Conclusions

The role of the immune system in tumor progression is the subject of study in leading laboratories around the world. The development of oncoimmunology and immunotherapy for tumors has revolutionized the treatment of cancerous diseases. Macrophages, natural killers, and T-cells play a central role in the destruction of tumor cells. The nature of the interaction between the tumor and its microenvironment is multifaceted. On one hand, tumor cells can reprogram immune competent cells and suppress their anti-tumor activity, while on the other hand, tumor cells can develop resistance to the cytotoxic effects of macrophages and other immune competent cells.

**Figure 1:**
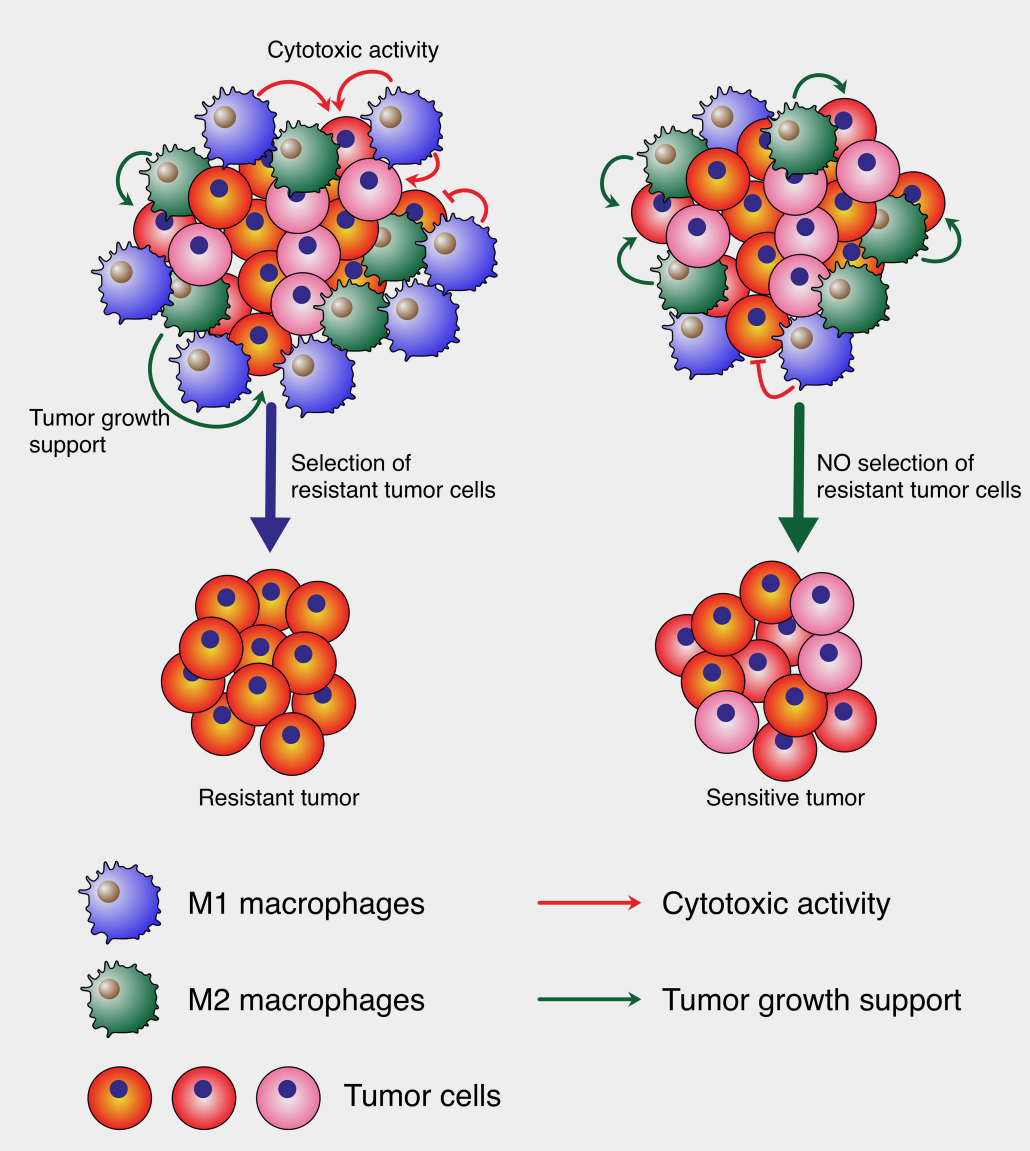
*Cytotoxic macrophage-mediated selection of tumor cells.* The left panel depicts a heterogeneous tumor microenvironment where large amount of M1 macrophages exert cytotoxic pressure (red arrows), leading to the elimination of sensitive tumor cells and the survival of resistant clones, culminating in the emergence of a resistant tumor. Concurrently, M2 macrophages support tumor growth (green arrows). In contrast, the right panel shows a balanced microenvironment where such selective pressure is low, allowing for the persistence of a mixed tumor cell population without the dominance of resistant clones, resulting in a sensitive tumor phenotype. This model illustrates how cytotoxic M1 can paradoxically contribute to tumor evolution.

As illustrated in **Figure 1**, M1 macrophages, despite their cytotoxic activity against tumor cells, can inadvertently drive tumor evolution by selecting for resistant cell populations. In a heterogeneous tumor microenvironment, M1 macrophages eliminate sensitive tumor cells, but their activity may leave behind and promote the expansion of resistant clones, resulting in a more aggressive tumor phenotype. This selection pressure ultimately leads to tumor relapse with enhanced resistance characteristics. In contrast, the absence of such selective pressure may preserve tumor cell sensitivity, as shown in the right panel of the figure. Here, the tumor retains a mixed population without the dominance of resistant phenotypes, underscoring the paradoxical role of cytotoxic macrophages in tumor progression.

In summary, recent years have provided compelling evidence for a new function of cytotoxic macrophages in tumors – namely, their ability to participate in the selection of more malignant tumor cells and to promote tumor progression. Current literature explains the minimal success of therapeutic strategies aimed at altering the phenotype of macrophages to cytotoxic. It is clear that there is a need to completely reconsider macrophage-mediated therapy strategies and adjust them, possibly by focusing on reducing the overall number of macrophages in malignant neoplasms.

The interaction between tumor cells and the immune system is complex and dynamic. As our understanding of this relationship deepens, it reveals that while immune cells are traditionally viewed as protectors against cancer, under certain conditions they can facilitate cancer adaptability and survival. This paradoxical behavior highlights the intricate balance of immune responses within the tumor microenvironment, where the same factors that are meant to fight the tumor can also end up supporting it. Thus, a nuanced approach is required in developing immune-based therapies, one that not only aims to activate immune responses but also precisely targets these responses to avoid unintended support of tumor growth and resistance. This ongoing research emphasizes the importance of developing targeted therapies that can selectively modulate the immune landscape of tumors, thereby enhancing the efficacy and specificity of cancer treatments.

## References

[1] Xu M Zhang T Xia R Wei Y Wei X. Targeting the tumor stroma for cancer therapy. Mol cancer. (2022) 21:208. doi: 10.1186/s12943-022-01670-1, PMID: 36324128 PMC9628074

[2] Li HX Wang SQ Lian ZX Deng SL Yu K. Relationship between tumor infiltrating immune cells and tumor metastasis and its prognostic value in cancer. Cells. (2022) 12:64. doi: 10.3390/cells12010064, PMID: 36611857 PMC9818185

[3] Finn OJ. Human tumor antigens yesterday, today, and tomorrow. Cancer Immunol Res. (2017) 5:347–54. doi: 10.1158/2326-6066.CIR-17-0112, PMID: 28465452 PMC5490447

[4] Sobhani N Tardiel-Cyril DR Davtyan A Generali D Roudi R Li Y. CTLA-4 in regulatory T cells for cancer immunotherapy. Cancers (Basel). (2021) 13:1440. doi: 10.3390/cancers13061440, PMID: 33809974 PMC8005092

[5] Sanchez-Reyes K Bravo-Cuellar A Hernandez-Flores G Lerma-Diaz JM Jave-Suarez LF Gomez-Lomeli P. Cervical cancer cell supernatants induce a phenotypic switch from U937-derived macrophage-activated M1 state into M2-like suppressor phenotype with change in Toll-like receptor profile. BioMed Res Int. (2014) 2014:683068. doi: 10.1155/2014/683068, PMID: 25309919 PMC4189768

[6] Yin T Fu CB Wu DD Nie L Chen H Wang Y. Apatinib suppressed macrophage-mediated Malignant behavior of hepatocellular carcinoma cells via modulation of VEGFR2/STAT3/PD-L1 signaling. Mol Biol (Mosk). (2023) 57:706–8. doi: 10.1134/S0026893323040180, PMID: 37528791

[7] Bingle L Brown NJ Lewis CE. The role of tumour-associated macrophages in tumour progression: implications for new anticancer therapies. JPathol. (2002) 196:254–65. doi: 10.1002/path.1027, PMID: 11857487

[8] Zhang QW Liu L Gong CY Shi HS Zeng YH Wang XZ. Prognostic significance of tumor-associated macrophages in solid tumor: a meta-analysis of the literature. PloS One. (2012) 7:e50946. doi: 10.1371/journal.pone.0050946, PMID: 23284651 PMC3532403

[9] Epelman S Lavine KJ Randolph GJ. Origin and functions of tissue macrophages. Immunity. (2014) 41:21–35. doi: 10.1016/j.immuni.2014.06.013, PMID: 25035951 PMC4470379

[10] Molawi K Wolf Y Kandalla PK Favret J Hagemeyer N Frenzel K. Progressive replacement of embryo-derived cardiac macrophages with age. J Exp Med. (2014) 211:2151–8. doi: 10.1084/jem.20140639, PMID: 25245760 PMC4203946

[11] Gratchev A Schledzewski K Guillot P Goerdt S. Alternatively activated antigen-presenting cells: molecular repertoire, immune regulation, and healing. Skin Pharmacol Appl skin Physiol. (2001) 14:272–9. doi: 10.1159/000056357, PMID: 11586068

[12] Mantovani A Locati M. Tumor-associated macrophages as a paradigm of macrophage plasticity, diversity, and polarization: lessons and open questions. Arteriosclerosis thrombosis Vasc Biol. (2013) 33:1478–83. doi: 10.1161/ATVBAHA.113.300168, PMID: 23766387

[13] Gratchev A Kzhyshkowska J Utikal J Goerdt S. Interleukin-4 and dexamethasone counterregulate extracellular matrix remodelling and phagocytosis in type-2 macrophages. Scandinavian J Immunol. (2005) 61:10–7. doi: 10.1111/j.0300-9475.2005.01524.x, PMID: 15644118

[14] Griffith TS Wiley SR Kubin MZ Sedger LM Maliszewski CR Fanger NA. Monocyte-mediated tumoricidal activity via the tumor necrosis factor-related cytokine, TRAIL. J Exp Med. (1999) 189:1343–54. doi: 10.1084/jem.189.8.1343, PMID: 10209050 PMC2193036

[15] Kovaleva OV Rashidova MA Samoilova DV Podlesnaya PA Tabiev RM Mochalnikova VV. CHID1 is a novel prognostic marker of non-small cell lung cancer. Int J Mol Sci. (2021) 22:450. doi: 10.3390/ijms22010450, PMID: 33466316 PMC7795388

[16] Nathan C. Role of iNOS in human host defense. Science. (2006) 312:1874–5. doi: 10.1126/science.312.5782.1874b, PMID: 16809512

[17] Mackaness GB. Cellular resistance to infection. J Exp Med. (1962) 116:381–406. doi: 10.1084/jem.116.3.381, PMID: 25240017

[18] Stein M Keshav S Harris N Gordon S. Interleukin 4 potently enhances murine macrophage mannose receptor activity: a marker of alternative immunologic macrophage activation. JExpMed. (1992) 176:287–92. doi: 10.1084/jem.176.1.287, PMID: 1613462 PMC2119288

[19] Wang N Liang H Zen K. Molecular mechanisms that influence the macrophage m1-m2 polarization balance. Front Immunol. (2014) 5:614. doi: 10.3389/fimmu.2014.00614, PMID: 25506346 PMC4246889

[20] Roszer T. Understanding the mysterious M2 macrophage through activation markers and effector mechanisms. Mediators inflammation. (2015) 2015:816460. doi: 10.1155/2015/816460, PMID: 26089604 PMC4452191

[21] Stifano G Christmann RB. Macrophage involvement in systemic sclerosis: do we need more evidence? Curr Rheumatol Rep. (2016) 18:2. doi: 10.1007/s11926-015-0554-8, PMID: 26700912

[22] Anders CB Lawton TM Smith HL Garret J Doucette MM Ammons MCB. Use of integrated metabolomics, transcriptomics, and signal protein profile to characterize the effector function and associated metabotype of polarized macrophage phenotypes. J leukocyte Biol. (2021) 111:667–93. doi: 10.1002/JLB.6A1120-744R, PMID: 34374126 PMC8825884

[23] Bai J Adriani G Dang T-M Tu T-Y Penny H-XL Wong S-C. Contact-dependent carcinoma aggregate dispersion by M2a macrophages via ICAM-1 and β2 integrin interactions. Oncotarget. (2015) 6:25295–307. doi: 10.18632/oncotarget.4716, PMID: 26231039 PMC4694832

[24] Edin S Wikberg ML Rutegard J Oldenborg PA Palmqvist R. Phenotypic skewing of macrophages *in vitro* by secreted factors from colorectal cancer cells. PloS One. (2013) 8:e74982. doi: 10.1371/journal.pone.0074982, PMID: 24058644 PMC3776729

[25] Brune B Dehne N Grossmann N Jung M Namgaladze D Schmid T. Redox control of inflammation in macrophages. Antioxid Redox Signal. (2013) 19:595–637. doi: 10.1089/ars.2012.4785, PMID: 23311665 PMC3718318

[26] Mauel J. Activation and cytotoxic activity of macrophages: a short review. Recent Results Cancer Res. (1976) 56:31–40. doi: 10.1007/978-3-642-81049-7_5, PMID: 794957

[27] De Groot JW De Weger RA Vandebriel RJ Den Otter W. Differences in the induction of macrophage cytotoxicity by the specific T lymphocyte factor, specific macrophage arming factor (SMAF), and the lymphokine, macrophage activating factor (MAF). Immunobiology. (1989) 179:131–44. doi: 10.1016/S0171-2985(89)80012-9, PMID: 2676851

[28] Chen S Saeed AFUH Liu Q Jiang Q Xu H Xiao GG. Macrophages in immunoregulation and therapeutics. Signal Transduction Targeted Ther. (2023) 8:207. doi: 10.1038/s41392-023-01452-1, PMID: 37211559 PMC10200802

[29] Petricevic B Laengle J Singer J Sachet M Fazekas J Steger G. Trastuzumab mediates antibody-dependent cell-mediated cytotoxicity and phagocytosis to the same extent in both adjuvant and metastatic HER2/neu breast cancer patients. J Trans Med. (2013) 11:307. doi: 10.1186/1479-5876-11-307, PMID: 24330813 PMC4029549

[30] Upton R Banuelos A Feng D Biswas T Kao K McKenna K. Combining CD47 blockade with trastuzumab eliminates HER2-positive breast cancer cells and overcomes trastuzumab tolerance. Proc Natl Acad Sci United States America. (2021) 118:e2026849118. doi: 10.1073/pnas.2026849118, PMID: 34257155 PMC8307693

[31] Ochoa MC Minute L Rodriguez I Garasa S Perez-Ruiz E Inoges S. Antibody-dependent cell cytotoxicity: immunotherapy strategies enhancing effector NK cells. Immunol Cell Biol. (2017) 95:347–55. doi: 10.1038/icb.2017.6, PMID: 28138156

[32] Zhang M Wen B Anton OM Yao Z Dubois S Ju W. IL-15 enhanced antibody-dependent cellular cytotoxicity mediated by NK cells and macrophages. Proc Natl Acad Sci United States America. (2018) 115:E10915–E24. doi: 10.1073/pnas.1811615115, PMID: 30373815 PMC6243244

[33] Gogesch P Dudek S van Zandbergen G Waibler Z Anzaghe M. The role of fc receptors on the effectiveness of therapeutic monoclonal antibodies. Int J Mol Sci. (2021) 22:8947. doi: 10.3390/ijms22168947, PMID: 34445651 PMC8396266

[34] Reis ES Mastellos DC Ricklin D Mantovani A Lambris JD. Complement in cancer: untangling an intricate relationship. Nat Rev Immunol. (2018) 18:5–18. doi: 10.1038/nri.2017.97, PMID: 28920587 PMC5816344

[35] Li L Yang H Li Y Li XD Zeng TT Lin SX. Hypoxia restrains the expression of complement component 9 in tumor-associated macrophages promoting non-small cell lung cancer progression. Cell Death Discov. (2018) 4:63. doi: 10.1038/s41420-018-0064-3, PMID: 29900010 PMC5992192

[36] Boehm U Klamp T Groot M Howard JC. Cellular responses to interferon-gamma. Annu Rev Immunol. (1997) 15:749–95. doi: 10.1146/annurev.immunol.15.1.749, PMID: 9143706

[37] Paradkar PH Mishra LS Joshi JV Dandekar SP Vaidya RA Vaidya AB. *In vitro* macrophage activation: A technique for screening anti-inflammatory, immunomodulatory and anticancer activity of phytomolecules. Indian J Exp Biol. (2017) 55:133–41., PMID: 30184414

[38] Djavaheri-Mergny M Amelotti M Mathieu J Besancon F Bauvy C Codogno P. Regulation of autophagy by NFkappaB transcription factor and reactives oxygen species. Autophagy. (2007) 3:390–2. doi: 10.4161/auto.4248, PMID: 17471012

[39] Yang YL Yang F Huang ZQ Li YY Shi HY Sun Q. T cells, NK cells, and tumor-associated macrophages in cancer immunotherapy and the current state of the art of drug delivery systems. Front Immunol. (2023) 14:1199173. doi: 10.3389/fimmu.2023.1199173, PMID: 37457707 PMC10348220

[40] Zhong Y Yi C. MicroRNA-720 suppresses M2 macrophage polarization by targeting GATA3. Biosci Rep. (2016) 36:e00363. doi: 10.1042/BSR20160105, PMID: 27354564 PMC4974597

[41] Ying H Kang Y Zhang H Zhao D Xia J Lu Z. MiR-127 modulates macrophage polarization and promotes lung inflammation and injury by activating the JNK pathway. J Immunol. (2015) 194:1239–51. doi: 10.4049/jimmunol.1402088, PMID: 25520401

[42] Boucher A Klopfenstein N Hallas WM Skibbe J Appert A Jang SH. The miR-23a approximately 27a approximately 24–2 microRNA Cluster Promotes Inflammatory Polarization of Macrophages. J Immunol. (2021) 206:540–53. doi: 10.4049/jimmunol.1901277, PMID: 33328213 PMC7855803

[43] Rodell CB Arlauckas SP Cuccarese MF Garris CS Li R Ahmed MS. TLR7/8-agonist-loaded nanoparticles promote the polarization of tumour-associated macrophages to enhance cancer immunotherapy. Nat Biomed Engineering. (2018) 2:578–88. doi: 10.1038/s41551-018-0236-8, PMID: 31015631 PMC6192054

[44] Liu L He H Liang R Yi H Meng X Chen Z. ROS-inducing micelles sensitize tumor-associated macrophages to TLR3 stimulation for potent immunotherapy. Biomacromolecules. (2018) 19:2146–55. doi: 10.1021/acs.biomac.8b00239, PMID: 29669207

[45] Shan H Dou W Zhang Y Qi M. Targeted ferritin nanoparticle encapsulating CpG oligodeoxynucleotides induces tumor-associated macrophage M2 phenotype polarization into M1 phenotype and inhibits tumor growth. Nanoscale. (2020) 12:22268–80. doi: 10.1039/D0NR04520A, PMID: 33146206

[46] Wei B Pan J Yuan R Shao B Wang Y Guo X. Polarization of tumor-associated macrophages by nanoparticle-loaded escherichia coli combined with immunogenic cell death for cancer immunotherapy. Nano Letters. (2021) 21:4231–40. doi: 10.1021/acs.nanolett.1c00209, PMID: 33998789

[47] Li J-X Shu N Zhang Y-J Tong Q-S Wang L Zhang J-Y. Self-assembled nanoparticles from the amphiphilic prodrug of resiquimod for improved cancer immunotherapy. ACS Appl Materials Interfaces. (2024) 16:25665–75. doi: 10.1021/acsami.4c01563, PMID: 38735053

[48] Lee J Im K-I Gil S Na H Min G-J Kim N. TLR5 agonist in combination with anti-PD-1 treatment enhances anti-tumor effect through M1/M2 macrophage polarization shift and CD8+ T cell priming. Cancer Immunology Immunother. (2024) 73:102. doi: 10.1007/s00262-024-03679-5, PMID: 38630304 PMC11024077

[49] Xiao H Guo Y Li B Li X Wang Y Han S. M2-like tumor-associated macrophage-targeted codelivery of STAT6 inhibitor and IKKβ siRNA induces M2-to-M1 repolarization for cancer immunotherapy with low immune side effects. ACS Cent Science. (2020) 6:1208–22. doi: 10.1021/acscentsci.9b01235, PMID: 32724855 PMC7379385

[50] Xia Q Tang Y Li W Liang T Zhou Y Liu J. Surface-engineered monocyte immunotherapy combined graphene quantum dots effective against solid tumor targets. Int J nanomedicine. (2023) 18:2127–40. doi: 10.2147/IJN.S404486, PMID: 37122502 PMC10145394

[51] Rao L Zhao SK Wen C Tian R Lin L Cai B. Activating macrophage-mediated cancer immunotherapy by genetically edited nanoparticles. Advanced materials. (2020) 32:e2004853. doi: 10.1002/adma.202004853, PMID: 33089578 PMC7686299

[52] Tang L Yin Y Cao Y Fu C Liu H Feng J. Extracellular vesicles-derived hybrid nanoplatforms for amplified CD47 blockade-based cancer immunotherapy. Advanced materials. (2023) 35:e2303835. doi: 10.1002/adma.202303835, PMID: 37384818

[53] Djureinovic D Weiss SA Krykbaeva I Qu R Vathiotis I Moutafi M. A bedside to bench study of anti-PD-1, anti-CD40, and anti-CSF1R indicates that more is not necessarily better. Mol cancer. (2023) 22:182. doi: 10.1186/s12943-023-01884-x, PMID: 37964379 PMC10644655

[54] Weiss SA Djureinovic D Jessel S Krykbaeva I Zhang L Jilaveanu L. A phase I study of APX005M and cabiralizumab with or without nivolumab in patients with melanoma, kidney cancer, or non–small cell lung cancer resistant to anti-PD-1/PD-L1. Clin Cancer Res. (2021) 27:4757–67. doi: 10.1158/1078-0432.CCR-21-0903, PMID: 34140403 PMC9236708

[55] Stone ML Herrera VM Li Y Coho H Yue Y Graham K. Abstract C029: Multiple non-redundant immune checkpoints direct therapeutic resistance to chemotherapy and anti-CSF1R in pancreatic ductal adenocarcinoma. Cancer Res. (2024) 84:C029–C. doi: 10.1158/1538-7445.PANCA2023-C029 PMC12560273 40920092

[56] Voissiere A Gomez-Roca C Chabaud S Rodriguez C Nkodia A Berthet J. The CSF-1R inhibitor pexidartinib affects FLT3-dependent DC differentiation and may antagonize durvalumab effect in patients with advanced cancers. Sci Trans Med. (2024) 16:eadd1834. doi: 10.1126/scitranslmed.add1834, PMID: 38266104

[57] Canli O Nicolas AM Gupta J Finkelmeier F Goncharova O Pesic M. Myeloid cell-derived reactive oxygen species induce epithelial mutagenesis. Cancer Cell. (2017) 32:869–83.e5. doi: 10.1016/j.ccell.2017.11.004, PMID: 29232557

[58] Moore RJ Owens DM Stamp G Arnott C Burke F East N. Mice deficient in tumor necrosis factor-alpha are resistant to skin carcinogenesis. Nat Med. (1999) 5:828–31. doi: 10.1038/10552, PMID: 10395330

[59] Noy R Pollard JW. Tumor-associated macrophages: from mechanisms to therapy. Immunity. (2014) 41:49–61. doi: 10.1016/j.immuni.2014.06.010, PMID: 25035953 PMC4137410

[60] Wang X Lin Y. Tumor necrosis factor and cancer, buddies or foes? Acta pharmacologica Sin. (2008) 29:1275–88. doi: 10.1111/j.1745-7254.2008.00889.x, PMID: 18954521 PMC2631033

[61] Landsberg J Kohlmeyer J Renn M Bald T Rogava M Cron M. Melanomas resist T-cell therapy through inflammation-induced reversible dedifferentiation. Nature. (2012) 490:412–6. doi: 10.1038/nature11538, PMID: 23051752

[62] Zhou X Hao Q Liao P Luo S Zhang M Hu G. Nerve growth factor receptor negates the tumor suppressor p53 as a feedback regulator. Elife. (2016) 5:e15099. doi: 10.7554/eLife.15099, PMID: 27282385 PMC4943851

[63] Das A Monteiro M Barai A Kumar S Sen S. MMP proteolytic activity regulates cancer invasiveness by modulating integrins. Sci Rep. (2017) 7:14219. doi: 10.1038/s41598-017-14340-w, PMID: 29079818 PMC5660204

[64] Wang XF Wang HS Wang H Zhang F Wang KF Guo Q. The role of indoleamine 2,3-dioxygenase (IDO) in immune tolerance: focus on macrophage polarization of THP-1 cells. Cell Immunol. (2014) 289:42–8. doi: 10.1016/j.cellimm.2014.02.005, PMID: 24721110

[65] Mellor AL Keskin DB Johnson T Chandler P Munn DH. Cells expressing indoleamine 2,3-dioxygenase inhibit T cell responses. J Immunol. (2002) 168:3771–6. doi: 10.4049/jimmunol.168.8.3771, PMID: 11937528

[66] Liu J Geng X Hou J Wu G. New insights into M1/M2 macrophages: key modulators in cancer progression. Cancer Cell Int. (2021) 21:389. doi: 10.1186/s12935-021-02089-2, PMID: 34289846 PMC8296555

[67] Boutilier AJ Elsawa SF. Macrophage polarization states in the tumor microenvironment. Int J Mol Sci. (2021) 22:6995. doi: 10.3390/ijms22136995, PMID: 34209703 PMC8268869

[68] Salmiheimo ANE Mustonen HK Vainionpää SAA Shen Z Kemppainen EAJ Seppänen HE. Increasing the inflammatory competence of macrophages with IL-6 or with combination of IL-4 and LPS restrains the invasiveness of pancreatic cancer cells. J Cancer. (2016) 7:42–9. doi: 10.7150/jca.12923, PMID: 26722359 PMC4679380

[69] Kuang D-M Zhao Q Peng C Xu J Zhang J-P Wu C. Activated monocytes in peritumoral stroma of hepatocellular carcinoma foster immune privilege and disease progression through PD-L1. J Exp Med. (2009) 206:1327–37. doi: 10.1084/jem.20082173, PMID: 19451266 PMC2715058

[70] Xie C Liu C Wu B Lin Y Ma T Xiong H. Effects of IRF1 and IFN-beta interaction on the M1 polarization of macrophages and its antitumor function. Int J Mol Med. (2016) 38:148–60. doi: 10.3892/ijmm.2016.2583, PMID: 27176664 PMC4899022

[71] Kainulainen K Takabe P Heikkinen S Aaltonen N de la Motte C Rauhala L. M1 macrophages induce protumor inflammation in melanoma cells through TNFR-NF-kappaB signaling. J Invest Dermatol. (2022) 142:3041–3051.e10. doi: 10.1016/j.jid.2022.04.024, PMID: 35580697

[72] Sharen G Cheng H Hu X Miao J Zhao D. M1−like tumor−associated macrophages enhance proliferation and anti−apoptotic ability of liver cancer cells via activating the NF−kappaB signaling pathway. Mol Med Rep. (2022) 26:331. doi: 10.3892/mmr.2022.12847, PMID: 36069218 PMC9494612

[73] Zhou Y Xia L Liu Q Wang H Lin J Oyang L. Induction of pro-inflammatory response via activated macrophage-mediated NF-kappaB and STAT3 pathways in gastric cancer cells. Cell Physiol biochemistry: Int J Exp Cell physiology biochemistry Pharmacol. (2018) 47:1399–410. doi: 10.1159/000490829, PMID: 29929193

[74] Lv C Li S Zhao J Yang P Yang C. M1 macrophages enhance survival and invasion of oral squamous cell carcinoma by inducing GDF15-mediated erbB2 phosphorylation. ACS Omega. (2022) 7:11405–14. doi: 10.1021/acsomega.2c00571, PMID: 35415372 PMC8992263

[75] Podlesnaya PA Kovaleva OV Petrenko AA Gratchev AN. Cytotoxic activity of macrophages as a tumor malignancy factor. Bull Exp Biol Med. (2022) 174:147–51. doi: 10.1007/s10517-022-05664-3, PMID: 36437331

[76] Kovaleva OV Podlesnaya PA Vasileva MV Kopnin PB Balkin AS Plotnikov AO. Transcriptome of lung cancer cells resistant to the cytotoxic activity of macrophages. Dokl Biochem Biophys. (2022) 507:312–7. doi: 10.1134/S160767292205009X, PMID: 36786993

[77] Li X Liu Y Wu B Dong Z Wang Y Lu J. Potential role of the OPG/RANK/RANKL axis in prostate cancer invasion and bone metastasis. Oncol Rep. (2014) 32:2605–11. doi: 10.3892/or.2014.3511, PMID: 25333856

[78] Tsukamoto S Ishikawa T Iida S Ishiguro M Mogushi K Mizushima H. Clinical significance of osteoprotegerin expression in human colorectal cancer. Clin Cancer research: an Off J Am Assoc Cancer Res. (2011) 17:2444–50. doi: 10.1158/1078-0432.CCR-10-2884, PMID: 21270110

[79] Ito R Nakayama H Yoshida K Kuraoka K Motoshita J Oda N. Expression of osteoprotegerin correlates with aggressiveness and poor prognosis of gastric carcinoma. Virchows Archiv: an Int J pathology. (2003) 443:146–51. doi: 10.1007/s00428-003-0845-8, PMID: 12838418

[80] Russmueller G Moser D Wurger T Wrba F Christopoulos P Kostakis G. Upregulation of osteoprotegerin expression correlates with bone invasion and predicts poor clinical outcome in oral cancer. Oral Oncol. (2015) 51:247–53. doi: 10.1016/j.oraloncology.2014.11.010, PMID: 25532817

[81] Chung ST Geerts D Roseman K Renaud A Connelly L. Osteoprotegerin mediates tumor-promoting effects of Interleukin-1beta in breast cancer cells. Mol cancer. (2017) 16:27. doi: 10.1186/s12943-017-0606-y, PMID: 28143606 PMC5286681

